# A randomized controlled trial of a senior centre group programme for increasing social support and preventing depression in elderly people living at home in Norway

**DOI:** 10.1186/1471-2318-12-20

**Published:** 2012-05-20

**Authors:** Hege Bøen, Odd Steffen Dalgard, Rune Johansen, Erik Nord

**Affiliations:** 1 Norwegian Institute of Public Health, Division of Mental Health, P.O. Box 4404 Nydalen, NO-0403, Oslo, Norway

**Keywords:** Elderly people, Social support, Depression, Prevention, Senior centre

## Abstract

**Background:**

Late-life depression is a common condition and a challenging public health problem. A lack of social support is strongly associated with psychological distress. Senior centres seem to be suitable arenas for community-based health promotion interventions, although few studies have addressed this subject. The objectives were to examine the effect of a preventive senior centre group programme consisting of weekly meetings, on social support, depression and quality of life.

**Methods:**

A questionnaire was sent to a random sample of 4,000 persons over 65 in Oslo, and a total of 2,387 completed questionnaires were obtained. These subjects served as a basis for recruitment of participants for a trial, with scores on HSCL-10 being used as a main inclusion criterion. A total of 138 persons were randomized into an intervention group (N = 77) and control group (N = 61). Final analyses included 92 persons. Social support (OSS-3), depression (BDI), life satisfaction and health were measured in interviews at baseline and after 12 months (at the end of the intervention programme). Perceptions of benefits from the intervention were also measured. Mean scores, SD, SE and CI were used to describe the changes in outcomes. Effect sizes were calculated based on the original scales and as Cohen’s d. Paired sample tests and ANOVA were used to test group differences.

**Results:**

There was an increase in social support in both groups, but greatest in the intervention group. The level of depression increased for both groups, but more so in the control than the intervention group. There was a decrease in life satisfaction, although the decrease was largest among controls. There were almost no differences in reported health between groups. However, effect sizes were small and differences were not statistically significant. In contrast, most of the participants said the intervention meant much to them and led to increased use of the centre.

**Conclusions:**

In all probability, the intervention failed to meet optimistic targets, but possibly met quite modest ones. Since intention-to-treat analysis was not possible, we do not know the effect on the intervention group as a whole. A further evaluation of these programmes is necessary to expand the group programme. For the depressed, more specialized programmes to cope with depression may be a more appropriate intervention.

**Trial Registration:**

DRKS00003120 on DRKS

## Background

Late-life depression and depressive symptoms are common conditions and a challenging public health problem. Studies from the Netherlands from 1999–2006 suggested a prevalence of 16% [1]. A Norwegian study reported that the prevalence of depression increased with age, and was highest among the eldest. Of those 80 years and older, depression was reported by 20% [2]. Depression among older people is often related to physical symptoms resulting from chronic diseases or other impairments [3], as the combination of chronic diseases and depressive conditions cause dramatic reductions in the quality of life [4,5].

A lack of social support is strongly associated with psychological distress in elderly people, and is an important psychosocial risk factor for depressive disorders later in life [6]. Hence, interventions targeting loneliness and social isolation seem to be a good strategy for prevention, which is also pointed out by Luanaigh and Lawlor in their review article on loneliness and the health of older people [7]. A systematic review conducted to examine the effects of health-promoting interventions identified nine of 10 effective interventions to alleviate social isolation and loneliness among older people. Most were group interventions with an educational or support input for specific groups of older people, and it appeared that programmes that enabled participants to be involved in planning, developing and delivering activities were most likely to be the most effective [8]. A randomized controlled trial showed a decrease in the feeling of loneliness among frail elders who had been exposed to a physically oriented rehabilitation programme [9].

The senior centre is the only welfare service in Norwegian elder care serving both fit and less functional pensioners over 65 years. Senior centres have the goal of maintaining physical and psychological activity, functional health, protection, in addition to the promotion of self-sufficiency and the prevention of psychosocial problems of loneliness and isolation in the elderly. They are organized as small local units for activity and social contact, have a small staff of 2–4 persons and are run in large part by volunteers; they can be characterized as a welfare service and a private responsibility, though not a statutory care service such as home help, home nursing and a residential care facility.

Senior centres seem to be suitable arenas for community-based health promotion interventions that target social isolation and loneliness. However, few studies have addressed this subject. Three studies located at senior centres with physical activity as the main outcome also report a preventive effect on psychological distress. One randomized controlled intervention study located at a senior centre with 201 frail older adults demonstrated significantly higher levels of physical activity and senior centre participation, as well as a significant reduction in the use of psychoactive medications in the intervention group [10]. Another randomized trial with 100 older adults in a senior centre showed that after six months the intervention group had significantly better scores on the Medical Outcomes Study Short Form (SF-36) health survey subscales, and fewer depressive symptoms than controls measured with the self-report depression scale CES-D [11]. A pre- test, post-test evaluation of a one-year prevention programme with the participation of 300 men and women aged 65 and older and 14 senior centres participating, concluded with a decrease in depression symptoms, better self-evaluated health and increased physical activity among the participants [12].

The purpose of the present study was to test the effects of a senior centre group programme on preventing depression, increasing social support and self-rated health and satisfaction with life. We hypothesized that the programme could cause lower score on a depression scale, and higher scores on life satisfaction, self-rated health and social support scales in the participants of the programme than in controls.

## The intervention

The intervention was initiated by the National Association for Public Health, which owns 32 senior centres in Norway, and sponsored by the Extra Foundation for Health and Rehabilitation. Its aims were to reach out to elderly people with symptoms of loneliness and some symptoms of psychological distress and also to increase the use of senior centres in a selection of elderly people in two districts of Oslo, Norway. By having these people participate in common senior centre activities, there was hope of increasing their feelings of social support, alleviating and preventing depression and increasing their satisfaction with life.

Independent of the location of the senior centres, there are number of studies which show that mental stimuli, social network and social engagement, nutrition intervention and physical activity have a positive effect on the mental health of the elderly [13-19]. The intervention programme, however, was based on practical experiences from senior centre leaders, as well as the goals of the senior centre and some Norwegian studies concerning the above mentioned themes (only in the Norwegian language) [20-23]. It was designed to produce practical knowledge how the senior centres could expand their activities.

**Figure 1:**
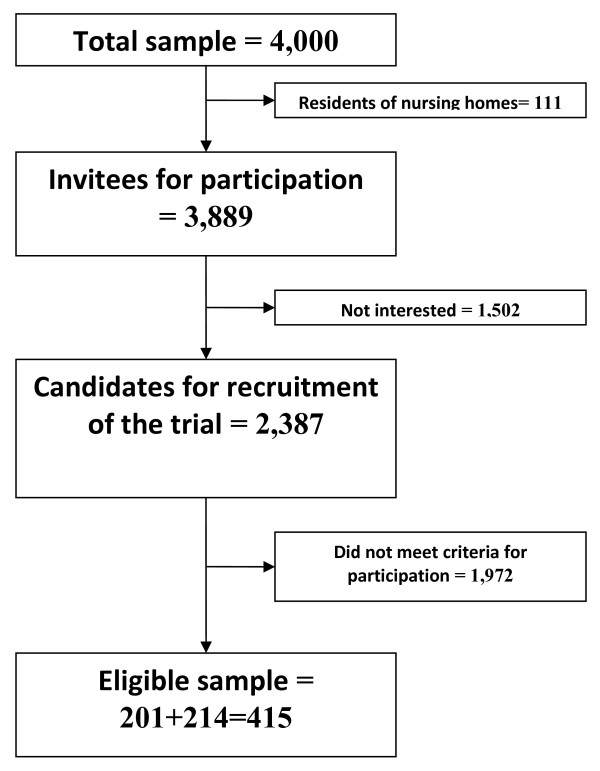
The process going from the gross sample to the eligible sample.

**Figure 2:**
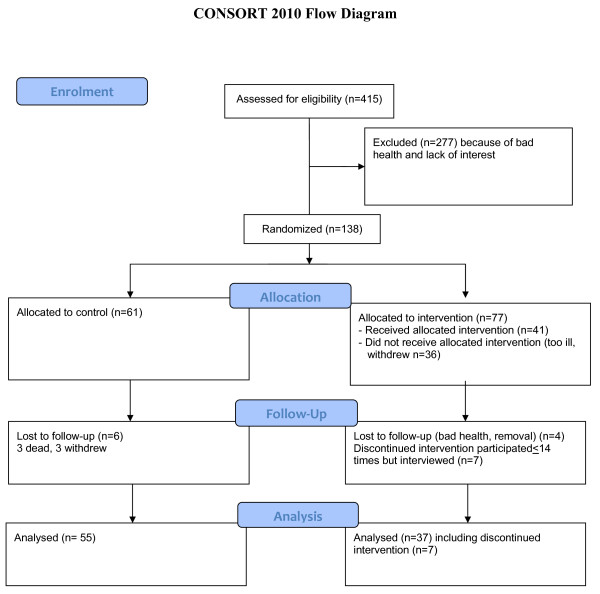
The process of going from the eligible sample to analysis.

The intervention was started in late January 2007, and was conducted in three senior centres in two municipal districts, with one in eastern Oslo and one in western Oslo. Elderly people who were eligible for participation (see Figures 1 and 2 in the Method section) were offered a programme consisting of a weekly group meeting of a three-hour duration that was carried out 35 to 38 times over the course of a year. Each group had a fixed membership and 7 – 10 participants. Addressing psychosocial problems such as depressive symptoms, loneliness and the isolation of elders within the senior centres context was chosen in this study because the senior centre leaders had a practical experience from local communities that many older persons were lonely and would benefit from a specially designed programme such as this. They also had a notion that many could not visit the centres due to a lack of transportation.

The intervention programme included transportation to and from the senior centre if needed and a warm meal at a low cost. A physical training programme developed by physiotherapists especially for older persons was included. It was easy to practice with a chair without changing clothes, footwear, etc. A self-help group discussed topics that the participants agreed upon themselves such as safety in the home and outdoors, how to avoid falling, social relations and aging, humour and laughter. These elements of the programme were well-known as key elements in daily activities at the centres. They were put together on the basis that if the participants who were slightly depressed when recruited would attend these groups, the content must be common and not too lengthy and that it was easy for them to meet. The group leaders were volunteers who had completed a training course for group leaders, and they were supervised by the project leader, who was a registered nurse and an experienced senior centre leader. A description of the introduction to the group methods and practical advice was created by the National Association of Public Health, both for the themes for the group discussion and for the physical training (only in the Norwegian language). The researchers had no part in planning or organizing this intervention.

The intervention was mostly implemented according to the project plan. Because the number of participants was reduced and the days available for the group programme had to fit the time schedules of the participants, the five groups were supplemented by other persons not taking part in the research project. In a logbook at each senior centre, the group leaders documented the names and dates of those present and absent from among the research participants.

The intervention was planned for 80 people, with this being the maximum number allowed by the resources available to the project and the three centres in question.

### Preliminary evaluation of the intervention

Prior to the outcome evaluation reported in this paper, the intervention was evaluated by the project leader with respect to the participant’s satisfaction. The participants were asked questions about health and well-being, and were tested for simple physical skills three weeks after the beginning of the programme and after 11 months. Both men and women reported being in a normal good mood after three weeks, though before the intervention they reported symptoms of distress and a lack of initiative. Some possible explanations for this are that the intervention had already fulfilled some of its intentions or that the participants reported what they thought the project leader expected. Among the women, 40% had made new friends, while the men reported no differences in friendships. Female participants also experienced that the number of visits from friends in their homes increased, and that the participants were active in the group setting and met on time. The project leader for the participants reported less loneliness, a better mood and better physical health [24].

### Main evaluation of the intervention

The present evaluation was conducted by researchers who had no part in planning or organizing the intervention. The evaluation was designed as a randomized controlled trial which aimed at comparing an intervention group of 80 people with an equally large control group. The organizers of the intervention cooperated with the evaluators in recruiting participants so that a sound, randomized controlled trial could be conducted. The study intervention did not require any large additional expenditure of resources, and was easily fitted into the ongoing senior centre programme.

We were not able to detect any controlled studies of the effect of the senior centres’ programmes on persons’ mental health, well-being and social support as main outcomes. It was hypothesized that the intervention was particularly suitable for the elderly with only slight depression, which affected the inclusion criteria, see below.

The limitation of the intervention to 80 people inevitably set a limit to the statistical power that would be possible to achieve in an evaluation. It was therefore acknowledged that an assessment of the effectiveness could lead to results with fairly wide margins of uncertainty, which would have to be read as indicative rather than conclusive. As shown below, some fairly clear conclusions can nonetheless be drawn.

## Methods

### Participants and recruitment

The study was approved by the Data Inspectorate and the National Committee for Medical and Health Research Ethics, (Southeast Region) in 2006.

The process of going from the gross random sample to the sample assessed for eligibility and randomization is shown in Figures 1 and 2.

As a first step towards selecting people for the trial, a random sample of 4,000 persons over the age of 65 years living at home - with 2,000 from each of the two municipal districts - was drawn from the Norwegian Population Register. Of these, 111 persons were residents of nursing homes and were excluded from the material.

Letters were sent to the remaining 3,889 persons in October 2006. The letter contained information about the senior centres and an extensive questionnaire for self-administration and postal return, and asked about gender, age, education, income, ethnicity, marital status, use of the senior centre, reason for non-use, functional impairments (physical and mental), social support and quality of life. One reminder was sent to those who did not respond. The completed questionnaires were scanned and quality controlled. A total of 2,387 out of the 3,889 persons (61%) were obtained as candidates for recruitment to the trial. The further details of those who did not answer are described in Bøen et al. [25].

For recruitment to the trial, an initial inclusion criterion (see below) was ‘having psychological distress according to Hopkins Symptom Checklist-10 (HSCL-10) in the range of 1.39 to 1.99’, which corresponds to ‘light depression’. Two other criteria were that the subjects should not have been regular users of the senior centre already, and that they wanted to be part of the current study. Of the 2,387 persons who responded satisfactorily to the questionnaire, 201 met these three initial eligibility criteria. The 201 were contacted by phone in order to make practical arrangements for face-to-face interviews in their homes. The purpose of the interviews was to make further observations regarding their eligibility and motivation for participation in the trial, and to obtain written informed consent. Specially trained social work students and retired social workers, researchers and project leaders conducted the interviews.

During this part of the recruitment process, a number of potential candidates dropped because of a lack of interest. The reasons given for this disinterest were bad health and a heavy burden of care. This was essentially as expected, although the *number* of dropouts was larger than anticipated and implied that the number of participants in the trial would be lower than planned. To avert a loss of statistical power beyond what had to initially be accepted for practical reasons, we carried out a second recruitment from the 2387 completed questionnaires, in which we expanded the inclusion criterion based on the HSCL-10 scale to the range from 1.20-3.90. This meant that we included candidates who were not depressed at all, although we also included candidates who were more depressed than in the first recruitment. Those who had answered less than seven out of 10 questions on HSCL-10 were excluded. An additional 214 persons were found eligible in this second round, yielding a total of 415 eligible persons. Persons in the second round were contacted and interviewed in their homes in the same way as the first 201 persons**.**

Figure 2 illustrate the process of going from the eligibility sample (415) to the randomized sample (138) and is explained in the following. The flow diagram (Figure 2) used is according to updated guidelines for reporting parallel group randomized trials [26,27].

In total, 277 of the 415 eligible persons dropped out, leaving 138 subjects for randomization in the trial, which was performed by stratifying many in the sample by geographical area and gender. Seventy-seven (55%) were allocated to the intervention group and 61 (45%) to the control group. A larger lot was drawn to the intervention group than to the control group because of an expected loss of participants.

The first group (those recruited on the basis of the initial HSCL criterion) started the intervention in January 2007, four weeks after baseline interviews with the outcome measures described below. Follow-up interviews took place in November/December of the same year.

The additional second group started the intervention in April 2007, four weeks after baseline interviews, with the follow-up interviews conducted in April/March 2008.

The control group was free to continue daily activities as they chose, and they were offered the same group activities as the intervention group after one year. They were not subjects of further follow-up studies after the intervention had ended.

### Data

One outcome parameter was social support, as measured by scores on the Oslo-3 Social support scale (OSS-3). The OSS-3 is based on three questions, with scores on each item and a sum score.

Oslo 1: How many people are you so close to that you can count on them if you have great personal problems? (none (1), 1–2 (2), 3–5 (3), 5+ (4))

Oslo 2: How much interest and concern do people show in what you do? (a lot (5), some (4), uncertain (3), little (2), none (1))

Oslo 3: How easy is it to get practical help from neighbours if you should need it? (very easy (5), easy (4), possible (3), difficult (2), very difficult (1))

In the present paper the sum score, ranging from 3 (worst) to 14 (best), is used. The OSS-3 has been used in several studies, thereby confirming its feasibility and predictive validity with respect to psychological distress [28,29].

Another outcome parameter was depression, as measured on the Beck Depression Inventory (BDI). The BDI is a 21-item, self-report scale, ranging from 0 (normal) to 3 (most severe), with a total maximum depression score of 63. This questionnaire is widely used among older adults, though not specifically developed for the geriatric population [30-33].

A third outcome parameter was life satisfaction, as measured by scores on a question about quality of life, ranging from 1–5, with 1 meaning very dissatisfied and 5 very satisfied.

A fourth outcome parameter was self-reported health, as measured by scores on a question of health, ranging from 1–4, with 1 meaning bad health and 4 very good health.

All of these measurements were conducted in an identical manner in face-to-face interviews, first at baseline and then at 12 months follow-up when the intervention had come to an end. After 12 months, the intervention group was also asked how much the weekly group programme meant to them, ranging from very much (4) to little (1). They were also asked if they had made any new friends or met the participants in a private setting (yes/no).

### Specific hypotheses to be tested

The expectation of those who initiated and organized the intervention was that it would increase the participants’ feelings of social support, alleviate and prevent depression and increase their satisfaction with life, though the exact strength of this expectation was not specified. We considered that the effects needed to be above a certain size to be clinically significant and thus of interest for policymaking. With respect to the Beck Depression Inventory, Bright et al. (1999) suggested that a change of at least 6 points is clinically significant, even though this judgement is somewhat arbitrarily related to the size of standard deviations commonly observed in BDI data. Nord and Dalgard conducted a cost-value analysis of a programme for coping with depression based on an observed effect of 3.3 points on the BDI [34]. This corresponded for instance to ‘somewhat less sadness, somewhat greater interest in others, somewhat greater enjoyment of activities and somewhat less problems with sleep’, which was clearly a significant difference. Consequently, we therefore lowered the requirement even further, and tested the hypothesis that the intervention yielded a mean effect of at least a 2 point improvement on the BDI. The implication was that if the effect was less than that, which we refer to as a ‘modest target’, the intervention may be difficult to justify. Since there was inevitably an element of subjectivity in such a choice of ‘satisfactory effect size’, we additionally applied a wider test criterion of 3 points on the BDI, which we refer to as an ‘optimistic target’.

We chose similar target levels of effect on the other three outcome measures. Here, we used the target level on the BDI as a reference and proportionally adjusted for differences in the length of the scales by looking at the standard deviations of scores on the four different scales as observed in the present study. As we shall see, the standard deviations were on the order of 6 for BDI, 2 for social support, 0.8 for life satisfaction and 0.6 for health. The target level of 2 points for BDI is 1/3 of the standard deviation. Applying the same fraction to the standard deviations of the other outcome measures, we achieved modest target levels of 0.67 points for social support, 0.27 points for life satisfaction and 0.2 points for health. The corresponding optimistic targets were 1.0, 0.4 and 0.3, respectively.

### Statistical methods and analyses

Baseline characteristics for the intervention and control groups were given as the percentage of distributions or mean values. Changes on the four outcome measures in the intervention and control groups were reported in terms of mean scores at intervention and at follow-up, including standard deviations (SD) at each of these, mean differences from intervention to follow-up, the standard deviations of these differences, the standard errors (SE) of the mean differences calculated as $SE=SD/\sqrt{N}$ and the 95% confidence intervals of the mean differences calculated as mean differences + / - 2 SE.

Effect sizes were first calculated in absolute terms as the *difference between the mean change* in the intervention group and the mean change in the control group on each of the four outcome measures, and second as a standardized effect size, which is the effect size divided by the mathematical mean of the standard deviations of the changes in the two groups (the Cohen’s d).

The standard error of an effect size in absolute terms on a particular outcome measure is calculated as the square root of the sum of the squared standard errors of the mean changes within the two groups.

We examined whether the estimated effect sizes in the study met the targets levels specified above by looking at the confidence intervals of the observed effects.

The Pearson’s correlation coefficient was used to describe the associations between the number of times participating in the group meetings and the outcome scores.

To be sure that the observed results were neither due to selection nor group differences, various raking techniques were used that are explained in the Discussion section.

To test differences at the group level, paired sample tests and a ‘one-way between groups’ ANOVA were used.

## Results

Of those allocated for intervention, 36 persons changed their minds about participating. They all cited too much stress or illness in their lives to carry through with the programme and completely withdrew from the study. Of those constituting the dropouts, one person died and some had great memory problems, which meant that they were not available for further interviews. In addition, four persons were lost to follow-up because of bad health and removal, which meant that a total of 40 persons dropped out (Figure 2).

Seven persons came to the group sessions 14 times or less and discontinued their participation, although group did not differ significantly from the others with respect to baseline characteristics.

Of those allocated for intervention, 37 (48%) took part in the follow-up, with the corresponding number of controls at 55 (90%). The total number of participants who completed the study was 92, which was much lower than what was initially hoped for (which was 80 for each group).

Ideally, analyses of interventions should be conducted on the basis of intention-to-treat. However, no data collection could be carried out with the 40 participants who were lost after the allocation and during follow up, due to their health status and withdrawal from the study. Their reasons for withdrawal were not questioned, and it would have been unethical to pressure them. The seven persons who did come to the group sessions 14 times or less (discontinued participation) were all followed up and interviewed at 12 months, and were included in the main analysis.

Characteristics of the study sample in the intervention and control groups at baseline are shown in Table 1:

**Table 1:** Characteristics of the sample

		**Intervention**	**Control**
**Gender**	Women	59.5	54.7
	Men	40.5	45.3
**Age group**	65-69	5.4	15.1
	70–79	35.1	35.8
	80+	59.5	49.1
Income*1	<150’	16.2	22.0
	150’-200’	27.0	28.0
	200’-300’	32.4	30.0
	300+	24.3	20.0
**Education**	Primary, 9 yrs	35.1	37.7
	Secondary,12 yrs	27.0	18.9
	College/University > 13 yrs	37.8	43.4
**Marital status**	Married/cohabiting	40.5	49.1
	Single	59.5	50.9
**District of town**	Ullern	56.8	64.2
	Østensjø	43.2	35.8
Life satisfaction* 2	Mean	3.65 (0.82)	3.84 (0.71)
Health* 3	Mean	2.44 (0.65)	2.55 (0.60)
Social support* 4	Mean	9.32 (2.01)	9.21 (2.00)
BDI*5	Mean	10.14 (6.63)	8.70 (4.85)

*1 Norwegian crowns.

*2 High value, most satisfied.

*3 High value, good health.

*4 High value, much support.

*5 High value, most depressed.

For categorical variables: Percentage; For continuous variables: Mean, Standard Deviations in brackets, total n = 92.

The intervention and control groups were fairly similar when compared at baseline for demographic characteristics, life satisfaction, health and social support, and the average age in the intervention group was slightly higher than among the controls. With respect to depression, the level of BDI indicated that the intervention group was slightly more depressed than the controls.

The scores on the outcome variables at baseline and the 12-month follow-up, as well as the difference scores for the intervention group and the control group, are shown in Table 2.

**Table 2:** Descriptive mean score, Standard deviations on the scales of life satisfaction, health, social support and Beck Depression Inventory at baseline and after 12 months, n total = 92 *

**Instruments and scoring range**	**Baseline**				**After 12 months**			**Differences Baseline-12 months**			**Effect size**
	**Groups**	**n**	**Mean score**	**S.D.**	**n**	**Mean score**	**S.D.**	**n**	**Mean change**	**S.D.**	**Cohen’s d**
**Life satisfaction**	Intervention	37	3.65	0.82	37	3.59	0.76	37	-0.06	0.78	0.22
**1-5**	Control	55	3.84	0.71	54	3.61	0.79	54	-0.22	0.74	
**Health**	Intervention	36	2.44	0.65	37	2.24	0.72	36	-0.20	0.74	0.07
**1-4**	Control	55	2.55	0.60	55	2.40	0.63	55	-0.15	0.52	
**Social support**	Intervention	37	9.32	2.02	37	9.97	2.05	37	0.65	1.46	0.12
**3-14**	Control	53	9.21	2.00	54	9.69	2.09	52	0.48	1.72	
**BDI**	Intervention	36	10.14	6.63	37	10.70	5.95	36	0.56	5.45	0.03
	Control	53	8.70	4.85	55	9.44	4.19	53	0.74	4.72	

* n differs, both in intervention group and in control group because not all the participants answered all the questions at both baseline and after 12 months.

For both groups, there was a decrease in life satisfaction, although the decrease was largest among controls. There were almost no differences in health between the groups. There was an increase in social support in both groups, but was greatest in the intervention group. For both groups the level of depression increased, but more so in the control than in the intervention group. Even so, the effect sizes were very small and all differences were far from being statistically significant. A null hypothesis – i.e. a hypothesis that the intervention in reality was without effect on the parameters in question – can by no means be rejected.

To look for a possible ‘dose-response’ effect, the outcome measures were correlated with the number of times participated in the group meetings. Both with respect to BDI, social support and health, improvements increased in step with the increasing number of times that the persons participated in the group meetings, whereas this was not the case for life satisfaction. Correlations were on the order of .1 - .2 (not shown in table), and none of them were significant.

Tables 3 and 4 show the development of BDI scores among those who were younger than 80 years and those who were 80 years and older.

**Table 3:** Development of Beck Depression Inventory score in intervention and control groups in 12 months for participants younger and older than 80 years (n = 92)

**Age**	**Younger than 80 years**				**80 years or above**			
**Time**	**T1**		**T2**		**T1**		**T2**	
	**intervention**	**control**	**intervention**	**control**	**intervention**	**control**	**intervention**	**control**
**Number of participants**	14	27	15	29	22	26	22	26
**BDI**	9.5	9.44	8.47	8.62	10.55	7.92	12.33	10.35

**Table 4:** Absolute and relative changes in Beck Depression Inventory score in intervention and control groups in 12 months for participants younger and older than 80 years (n = 92)

**Age**	**Younger than 80 years**		**80 years or above**	
	**intervention**	**control**	**intervention**	**control**
**Changes in BDI** (absolute)	- 1.03	- 0.82	+ 1.68	+ 2.43
**Changes in BDI** (relative)	- 10.8%	- 8.7%	+ 19.9%	+ 30.7%

There was no significant difference between the intervention and control groups in any of the two age groups.

A comparison with memory impairments revealed no significant differences between the intervention group and the control group, either for participants younger or older than 80 at time t2. There was no significant difference with respect to memory impairment at t2 between the two age groups (not shown in table). The figures were too small to test for use of medication against depression among the participants.

Table 5 shows the effect estimates compared to the previously chosen clinically significant target levels.

**Table 5:** Effect estimates of life satisfaction, health, social support and Beck Depression Inventory compared to reasonable, clinically significant target levels

	**Effect estimate absolute**	**SE**	**95% CI**	**Target level**	
	**Mean**			**Modest**	**Optimistic**
**Life satisfaction**	0.17	0.16	−0.15, +0.49	0.27	0.40
**Health**	−0.05	0.14	−0.33, +0.23	0.20	0.30
**Social support**	0.17	0.32	−0.47, +0.81	0.67	1.00
**BDI**	0.18	1.12	−2.06, +2.42	2.00	3.00

For social support, health and life satisfaction, the ‘optimistic’ target levels were higher than the upper limit of the 95% confidence intervals for the effect estimates, while for BDI, the optimistic target level was in the upper tail. Hence, the data strongly suggest the rejection of a hypothesis that the intervention yielded large effects. In contrast, the ‘moderate’ target levels were inside the effect size confidence intervals on all four parameters, meaning that the possibility of moderate effects (as defined above) cannot be rejected.

The participants in the research intervention group were also asked after 12 months to evaluate how much this intervention had meant to them. Of the 37 participants, 19 answered that it meant very much or much, 13 persons answered some and five persons said that it meant little. The ANOVA ‘one-way between groups’ analysis carried out with social support as the dependent variable suggested that those who valued the meetings as most meaningful also experienced the most improvement in social support, which was nearly significant (p < 0.08). Half the intervention group had made new friends, and 25 persons now availed themselves of more of the activities at the senior centre (not shown in the table).

## Discussion

The strength of this study is that it has a randomized, controlled design and uses well-established and validated outcome measures for social support and depression. Its main weaknesses are the high percentage of dropouts, which may have led to a selection bias by making it difficult to do a completely fair comparison between the groups (cf. the note above about the intention-to-treat analysis).

One possible explanation for the loss of participants in the intervention group after the randomization could be that when it came closer to the start of the programme, the participants started to have second thoughts and the obligation to weekly meetings for a year seemed too much to fulfil. As a result, the easiest way out was to withdraw at an early stage. The controls accepted three home visits during the year and a coming programme the next year, which did not imply that much personal commitment. To cushion this effect, we could have followed up the participants more closely by, e.g. phone calls.

The low number of participants, which leaves the study with a low statistical power, is also a problem. Both of these problems were difficult to avoid given the limited resources available in the intervention project, which calls for great care when interpreting the findings.

There were some differences between the intervention and control groups, both with respect to size and various socio-economic variables, and at baseline and the end of the intervention, thereby suggesting that the observed results might have been due to the selection mechanisms on one or more variable(s). However, including the variables under consideration as predictors in regression analyses, did not significantly affect the results, which suggest that the observed results in the present paper were neither due to selection nor group differences.

This was also confirmed by using various raking techniques [35,36]. More specifically, we first assumed that both the intervention and control group were identically distributed with respect to the different variables from Table 1: gender, age, income, education, marital status and geography at baseline, although this did not significantly affect the BDI level. Moreover, since the dropout rate from baseline to the end of the intervention was different in the two groups, we also checked whether this could affect the change in BDI from baseline to the end of the intervention by applying similar techniques. However, this did not significantly affect the BDI levels.

The effect of dropouts is difficult to judge. There are arguments for assuming that the dropouts would have benefited less than those who stayed, so that the inclusion of the dropouts would have made the differences between the intervention group and the control group smaller. But the opposite is also conceivable. Dropouts reported higher levels of stress and illness, so perhaps socializing at senior centres would have been of particular help to these people.

Disregarding the possible biases related to the high dropout rate, we may draw some conclusions from the data. Since all 95% confidence intervals of effect estimates clearly overlap with zero, it is impossible to reject the null hypothesis (that the intervention did not have any effect). That does not in itself mean that the intervention *cannot* have been effective, as there may have been positive effects that just do not show up in a statistically significant way due to the small sample size. We therefore focused on the possibility of there being more than just small and perhaps clinically insignificant effects, i.e. the possibility of effects that one would reasonably consider to be of clinical and policymaking interest. From this perspective, we suggested specific target values for effect sizes which the intervention should have been able to meet. According to our data, the intervention in all probability failed to meet optimistic targets, but possibly met quite modest ones. The latter possibility is supported by the positive reporting from participants with respect to satisfaction with the intervention. There was also a tendency towards a ‘dose–response’ effect, although this was not significant.

The very modest effect observed on BDI was somewhat surprising. An important concern is whether BDI is an inappropriate instrument in this context, in which the majority were over 80 years old. This inventory was chosen because it is widely used among older adults, with a well-documented high reliability, internal consistency and validity. The BDI also demonstrates a good discrimination between patients with varying degrees of depression, and accurately reflects changes in the intensity of depression over time [30-33]. Still, it may be difficult to separate depression and cognitive impairments, even in a diagnostic evaluation [37]. Since the majority of participants were over the age of 80 years, and knowing that the incidence of cognitive decline increases sharply in this age group, stratification in age groups over and less than 80 years was conducted. There were no significant differences in BDI scores between the intervention and control groups in either of the two age groups. Furthermore, no significant differences were found in memory impairment.

There was a relatively large but non-significant decrease in BDI score among persons aged less than 80 years, and a significant increase in BDI score among persons over 80 years. It could well be that participants from 65 to 79 experienced a process of awareness and optimism as a result of being surveyed. In contrast, the oldest group might have experienced no awareness and optimism due to their age and future expectations from the staring point to the end of the intervention.

Another possible explanation for the modest effect on BDI could be that the level of depression in the sample was too low (a mean BDI score at baseline of approximately 10) for a substantial effect to be expected from the intervention, though this explanation is not supported by a subgroup analysis of the data. When we split the material into those with a BDI score equal to or less than 10 and those with a BDI score higher than 10 (not reported in the tables), we observe that the positive effect is almost statistically significantly *smaller* in the high BDI group than in the low BDI group.

An alternative explanation is therefore that an intervention of this type does not so much serve to improve the condition of those who already have considerable depression, but rather to avert development of more severe depression in those who have only mild symptoms.

With regard to the fact that the intervention programme at most had a modest effect upon depression, the frequency of meetings and the level of competence of the group leaders must be taken into consideration in the evaluation of this programme. The leaders were volunteers and had no health professional or social work background that qualified them to address mental health problems, and most of them had no prior experience with conducting group programmes. If this programme is meant to address users’ special mental challenges, more experienced and professional group leaders are needed. The substance of the programme must then be developed towards a treatment course, such as a Coping With Depression Course (CWD) for the elderly. An effectiveness trial proved the CWD course to be effective for older people with subclinical depression, as well as for those with a current major depression [38].

## Conclusions

According to our data, the intervention in all probability failed to meet optimistic targets, but a ‘modest effect’ cannot be rejected. The latter possibility of a modest effect is supported by the positive reporting with respect to satisfaction with the intervention and a tendency toward a dose–response effect from participants who stayed in the group until the study period ended. Since the intention-to-treat analysis was not possible, we do not know the effect of the programme on the intervention group as a whole. A further evaluation of these programmes is necessary to expand the group programme. For the depressed, more specialized programmes to cope with depression may be a more appropriate intervention.

## Competing interests

None of the authors had any economic interest in the project.

## Author’s contributions

HB and OSD were responsible for the design. EN contributed to determining sample size. HB was responsible for the data collection. RJ performed the randomization. HB, OSD and RJ conducted the data analysis. HB and OSD were responsible for drafting the manuscript. RJ commented on the possibility of a selection bias. EN made extensive amendments and revisions for important substantial content. OSD supervised the study. All authors read and approved the final manuscript.

## Pre-publication history

The pre-publication history for this paper can be accessed here:

http://www.biomedcentral.com/1471-2318/12/20/prepub

## References

[B1] Smit F Ederveen A Cuijpers P Deeg D Beekman A Opportunities for cost-effective prevention of late-life depression: an epidemiological approach Arch Gen Psychiatry 2006 63 290 296 10.1001/archpsyc.63.3.290 16520434

[B2] Stordal E Bjartveit KM Dahl NH Kruger O Mykletun A Dahl AA Depression in relation to age and gender in the general population: the Nord-Trondelag Health Study (HUNT) Acta Psychiatr Scand 2001 104 210 216 10.1034/j.1600-0447.2001.00130.x 11531658

[B3] Stordal E Bjelland I Dahl AA Mykletun A: Anxiety and depression in individuals with somatic health problems. The Nord-Trondelag Health Study (HUNT) Scand J Prim Health Care 2003 21 136 141 10.1080/02813430310002030 14531503

[B4] Wittchen HU Jacobi F Size and burden of mental disorders in Europeùa critical review and appraisal of 27 studies Eur Neuropsychopharmacol 2005 15 357 376 10.1016/j.euroneuro.2005.04.012 15961293

[B5] Üstün TB Ayuso-Mateos JL Chatterji S Mathers C Murray CJL Global burden of depressive disorders in the year 2000 Br J Psychiatry 2004 184 386 10.1192/bjp.184.5.386 15123501

[B6] Bruce ML Psychosocial risk factors for depressive disorders in late life Biol Psychiatry 2002 52 175 184 10.1016/S0006-3223(02)01410-5 12182924

[B7] Luanaigh CË Lawlor BA Loneliness and the health of older people Int J Geriatr Psychiatry 2008 23 1213 1221 10.1002/gps.2054 18537197

[B8] Cattan M White M Bond J Learmouth A Preventing social isolation and loneliness among older people: a systematic review of health promotion interventions Ageing and society 2005 25 41 67 10.1017/S0144686X04002594 27736564

[B9] Ollonqvist K Palkeinen H Aaltonen T Pohjolainen T Puukka P Hinkka K Alleviating loneliness among frail older people Findings from a randomised controlled trial The International Journal of Mental Health Promotion 2008 10 26 34

[B10] Leveille SG Wagner EH Davis C Grothaus L Wallace J LoGerfo M Preventing disability and managing chronic illness in frail older adults: A randomized trial of a community-based partnership with primary care J Am Geriatr Soc 1998 46 1191 9777899 10.1111/j.1532-5415.1998.tb04533.x

[B11] Wallace JI Buchner DM Grothaus L Leveille S Tyll L LaCroix AZ Implementation and effectiveness of a community-based health promotion program for older adults The Journals of Gerontology: Series A 1998 53 M301 10.1093/gerona/53a.4.m301 18314570

[B12] Phelan EA Williams B Leveille S Snyder S Wagner EH LoGerfo JP Outcomes of a community-based dissemination of the Health Enhancement Program J Am Geriatr Soc 2002 50 1519 1524 10.1046/j.1532-5415.2002.50407.x 12383149

[B13] Barnett PA Gotlib IH Psychosocial Functioning and Depression - Distinguishing Among Antecedents, Concomitants, and Consequences Psychol Bull 1988 104 97 126 3043529 10.1037/0033-2909.104.1.97

[B14] Beekman ATF Penninx B Deeg DJH Ormel J Braam AW Van Tilburg W Depression and physical health in later life: results from the Longitudinal Aging Study Amsterdam (LASA) J Affect Disord 1997 46 219 231 10.1016/S0165-0327(97)00145-6 9547118

[B15] Fratiglioni L Winblad B von Strauss E Prevention of Alzheimer's disease and dementia, Major findings from the Kungsholmen Project Physiol Behav 2007 92 98 104 10.1016/j.physbeh.2007.05.059 17588621

[B16] Golden J Conroy RM Bruce I Denihan A Greene E Kirby M Loneliness, social support networks, mood and well-being in community-dwelling elderly Int J Geriatr Psychiatry 2009 24 694 700 10.1002/gps.2181 19274642

[B17] Kawachi I Berkman LF Social ties and mental health J Urban Health 2001 78 458 467 10.1093/jurban/78.3.458 11564849 PMC3455910

[B18] Mowe M Bohmer T Kindt E Reduced nutritional status in an elderly population (> 70 y) is probable before disease and possibly contributes to the development of disease [published erratum appears in Am J Clin Nutr 1994 Aug; 60 (2): 298] Am J Clin Nutr 1994 59 317 8310980 10.1093/ajcn/59.2.317

[B19] Vink D Aartsen MJ Schoevers RA Risk factors for anxiety and depression in the elderly: A review J Affect Disord 2008 106 29 44 10.1016/j.jad.2007.06.005 17707515

[B20] Pettersen AM Laake K Hvem bruker eldresenteret? hva er viktig for å ta senteret i bruk? Oslo Nasjonalforeningens forskergruppe i geriatri Universitetsseksjonen 2000 Geriatrisk avdeling, Ullevål sykehus

[B21] Pettersen AM Laake K Hukommelsesvansker, angst og depresjon hos hjemmeboende eldre Oslo: Nasjonalt kompetansesenter for aldersdemens Nasjonalforeningens forskergruppe i geriatri 2003

[B22] Sommerfeldt E Bevefelt E Eldresentre i utvikling 1993 Oslo: Norsk Gerontologisk Institutt

[B23] Thorsen K Levekår, ressurser og bruk av eldresenter 1983 Oslo: Norsk Gerontologisk Institutt

[B24] Renskaug IT Rehablitering i eldre senter. Rehabilitation in a senior centre 2008 2005/3/0266 edition: Nasjonalforenigen for folkehelsen

[B25] Boen H Dalgard OS Johansen R Nord E Socio-demographic, psychosocial and health characteristics of Norwegian senior centre users: A cross-sectional study Scand J Public Health 2010 10.1177/1403494810370230 20484305

[B26] Schulz K Altman D Moher D CONSORT 2010 Statement: updated guidelines for reporting parallel group randomised trials BMC Med 2010 8 18 10.1186/1741-7015-8-18 20334633 PMC2860339

[B27] Moher D Hopewell S Schulz KF Montori V Gotzsche PC Devereaux PJ CONSORT 2010 Explanation and Elaboration: updated guidelines for reporting parallel group randomised trials Br Med J 2010 340 c869 10.1136/bmj.c869 20332511 PMC2844943

[B28] European Union Public Health Information System 2009 Dalgard OS: Social support: occurence

[B29] Korkeila J Lehtinen V Bijl R Dalgard OS Kovess V Morgan A Establishing a set of mental health indicators for Europe Scand J Public Health 2003 31 451 459 10.1080/14034940210165208 14675937

[B30] Burns A Lawlor B Craig S Assessment scales in old age psychiatry Aust N Z J Psychiatry 2000 34 888 889

[B31] Beck AT Ward CH Mendelson ML Mock JE Erbaugh JK An inventory for measuring depression Arch Gen Psychiatry 1961 4 561 10.1001/archpsyc.1961.01710120031004 13688369

[B32] Beck RA Aaron T Psychometric properties of the Beck Depression Inventory: Twenty-five years of evaluation Clin Psychol Rev 1988 8 77 100 10.1016/0272-7358(88)90050-5

[B33] Richter P Werner J Heerlein A Kraus A Sauer H On the validity of the Beck Depression Inventory Psychopathology 2000 31 160 168 9636945 10.1159/000066239

[B34] Nord E Dalgard OS Economic evaluation of a course in coping with depression Tidsskr Nor Laegeforen 2006 126 586 588 16505865

[B35] Battaglia MP Izrael D Hoaglin DC Frankel MR Tips and tricks for raking survey data (aka sample balancing) Abt Associates 2004 4740 4744

[B36] Johansen R Rognerud M Sundet JM Aarø LE Observed Trends in Mental Health - a Strategy to Adjust for Nonresponse Bias and Demographic Changes in Survey Data 2010 submitted paper 10.1177/1403494812461732 23051586

[B37] Blazer DG Depression in late life: review and commentary Focus 2009 7 118 10.1093/gerona/58.3.m249 12634292

[B38] Haringsma R Engels GI Cuijpers P Spinhoven P Effectiveness of the Coping With Depression (CWD) course for older adults provided by the community-based mental health care system in the Netherlands: a randomized controlled field trial Int Psychogeriatr 2005 18 307 325 16255838 10.1017/S104161020500253X

